# Near‐Field Optical Nanopatterning of Graphene

**DOI:** 10.1002/smsc.202500184

**Published:** 2025-06-30

**Authors:** Gour Mohan Das, Eero Hulkko, Pasi Myllyperkiö, Andreas Johansson, Mika Pettersson

**Affiliations:** ^1^ Nanoscience Center Department of Chemistry University of Jyväskylä P. O. Box 35 FI‐40014 Jyväskylä Finland; ^2^ Nanoscience Center Department of Biological and Environmental Science University of Jyväskylä P. O. Box 35 FI‐40014 Jyväskylä Finland; ^3^ Nanoscience Center Department of Physics University of Jyväskylä P. O. Box 35 FI‐40014 Jyväskylä Finland

**Keywords:** direct femtosecond laser writing, graphene, nano‐Fourier transform infrared spectroscopy, near‐field nanopatterning, scattering‐type scanning near‐field optical microscopy

## Abstract

2D materials are emerging as transformative platforms for next‐generation memory, sensing, photonic, and quantum devices due to their extraordinary optical, mechanical, and electronic properties. A key challenge is achieving controlled and precise nanopatterning to unlock tailored functionalities. This work uses a direct laser writing method to introduce a near‐field‐mediated nanopatterning technique that delivers ≈10–30 nm lateral and sub‐5 nm vertical modification on graphene under ambient conditions. This approach uses a pulsed femtosecond laser in the visible wavelength range with scattering‐type scanning near‐field optical microscopy (s‐SNOM), where the s‐SNOM tip serves as a nanoscale probe. The resultant nanopatterns exhibit highly symmetric, periodic nanoscale holes with spherical perforations (nanopunch holes), with 5–25 nm dimensions. Importantly, nano‐ Fourier transform infrared spectroscopy reveals selective oxidative functionalization at the periphery of the nanopunch holes, highlighting a controlled surface modification of graphene. By finely tuning experimental parameters such as laser exposure time, the nanopatterning feature size ranging from 1–30 nm, and the resulting shapes from nanoscale elevated structures (nanoblister shape) to punched holes can be precisely modulated. This nanopatterning strategy achieves feature sizes at the sub‐10 nm scale and represents an advancement toward fabricating all‐2D material devices, setting new benchmark in nanoscale manufacturing for quantum and photonic technologies.

## Graphical abstract

Scattering‐type scanning near‐field optical microscopy‐driven near‐field optical nanopatterning of graphene is studied.

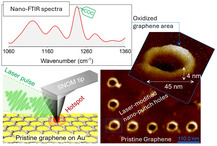

## 1 Introduction

The unique physicochemical properties and atomically thin structure of graphene and other 2D materials have made them a focal point of contemporary research and technological innovations.^[^ ^1^, ^2^ ^]^ These 2D materials have huge potential across a range of applications, including photonics,^[^ ^3^ ^]^ electronics,^[^ ^4^ ^]^ catalysis,^[^ ^5^ ^]^ biosensing,^[^ ^6^ ^]^ and emerging fields^[^ ^2^ ^]^ such as neuromorphic computing and quantum technologies. However, unlocking the maximum capabilities of these quantum‐confined 2D materials^[^ ^7^ ^]^ requires precise control over the structure and nanoscale properties. Thus, nanoscale patterning stands out as a vital technology for several reasons: creating new functionalities, ensuring seamless device integration, performance enhancement, scalability, and controlled and high‐precise fabrication.^[^ ^8^ ^]^ 2D materials with nanoscale patterning have a wide range of applications, including electronics,^[^ ^9^, ^10^ ^]^ sensors,^[^ ^11^ ^]^ energy devices,^[^ ^12^ ^]^ photonics,^[^ ^13^ ^]^ optoelectronics,^[^ ^14^ ^]^ biotechnology,^[^ ^15^ ^]^ and environmental technology.^[^ ^16^ ^]^ Traditional techniques such as electron beam lithography (EBL)^[^ ^17^ ^]^ and reactive ion etching (RIE)^[^ ^18^ ^]^ methods are mostly used for creating nanometer‐scale patterns on 2D materials (comparison between EBL and RIE is given in section B, Supporting Information). However, RIE can be harmful to atomically thin flakes,^[^ ^19^ ^]^ whereas EBL is regarded as a complicated process because of its high instrumentation costs, the need for high‐vacuum conditions, intricate operational procedures, and limited throughput.^[^ ^20^ ^]^ Laser‐based fabrication methods, which are classified as optical patterning techniques for 2D materials, have emerged as a very promising alternative, providing high‐throughput, adaptable, and in situ nanopatterning capabilities via precise laser beam control.^[^ ^19^, ^20^, ^21^, ^22^ ^]^ State‐of‐the‐art optical patterning techniques for 2D materials primarily rely on mechanisms such as site‐specific control over material thickness through laser thinning,^[^ ^23^ ^]^ laser‐induced doping,^[^ ^24^ ^]^ phase transitions,^[^ ^25^ ^]^ selective oxidation triggered by laser irradiation,^[^ ^26^ ^]^ and laser ablation for patterning.^[^ ^27^ ^]^ These techniques allow for a broad range of laser sources, from high‐power pulsed lasers to low‐power continuous lasers, offering flexibility in adjusting timescales and laser intensity to suit specific applications.^[^ ^20^ ^]^ The ability to fine‐tune these parameters makes laser‐assisted technologies a promising alternative to traditional lithographic and chemical methods for processing and functionalizing 2D materials. However, the spatial resolution of laser‐processing methods is usually diffraction‐limited to about 250 nm, which limits possible applications. Thus, it is important to develop subdiffraction‐limited super‐resolution techniques, which can bring capabilities for optical techniques to match EBL performance in spatial resolution.

Laser processing provides a versatile platform for patterning and modifying 2D materials with high precision, eliminating the need for masks or photoresists and thereby minimizing the risks of contamination and chemical damage. State‐of‐the‐art super‐resolution optical nanopatterning, particularly direct laser writing techniques, such as stimulated emission depletion,^[^ ^28^ ^]^ two‐photon polymerization,^[^ ^29^ ^]^ maskless optical projection nanolithography,^[^ ^30^ ^]^ and peripheral photoinhibition direct laser writing,^[^ ^31^ ^]^ have surpassed the optical diffraction limit, achieving feature sizes below 100 nm. Recent studies have explored direct writing technologies for nanopatterning, primarily using probes and lasers as key tools. While probe‐based and laser‐based nanofabrication have been widely reported, this study focuses on the synergistic use of both. Specifically, we explore examples of near‐field nanopatterning driven by laser‐coupled scanning near‐field optical microscopy (SNOM) (all near‐field nanopatterning techniques reported in the literature are listed in Table 2, given in Supporting Information). Near‐field two‐photon lithography, using 120 fs laser pulses at 790 nm in an apertureless SNOM, has demonstrated 70 nm resolution.^[^ ^32^ ^]^ Sub‐30 nm lithography has been achieved by combining near‐field scanning optical microscopy with femtosecond laser pulses.^[^ ^33^ ^]^ The integration of probe lithography, two‐photon lithography, and bowtie apertures has enabled nanoscale lines as small as 24 nm in photoresist.^[^ ^34^ ^]^ Lu et al. reported nanopits of 28–40 nm on gold films using a Q‐switched neodymium‐doped yttrium aluminum garnet (Nd:YAG) laser focused on a silicon tip, driven by near‐field enhancement, and mechanical indentation.^[^ ^35^ ^]^ Milner et al. demonstrated floating tip nanolithography for surface modification of polymers and metals with a spatial resolution of ≈20 nm, without direct contact.^[^ ^36^ ^]^ Yin et al. reported the controllable fabrication of sub‐20 nm structures via a coupling field tip‐enhancement effect induced by nanosecond pulsed laser irradiation.^[^ ^37^ ^]^ Our group has also contributed to the field of optical patterning techniques for 2D materials, particularly through femtosecond laser‐induced two‐photon oxidation of graphene.^[^ ^26^, ^38^, ^39^, ^40^, ^41^, ^42^, ^43^, ^44^ ^]^ This approach enables precise control over oxidative functionalization levels by adjusting the intensity and dose of laser irradiation, leading to the formation of patterned graphene oxide regions. By precisely adjusting the site and duration of the laser‐graphene interaction, continuous tuning of oxidation was achieved, allowing for the introduction of a bandgap and modification of both electrical and optical properties.

In this manuscript, a new apertureless direct laser writing technique for nanopatterning of graphene surfaces under ambient conditions is introduced by combining a femtosecond pulsed laser with scattering‐type SNOM (s‐SNOM). The key advancement is the use of the s‐SNOM tip for two‐photon oxidation of graphene nanopatterning. Near‐field generated at the tip apex drives the patterning process, enabling precise control at the nanoscale. Symmetrical, periodic, and reproducible nano‐punch holes (circular nanoscale hole patterns with surrounding elevated rings) were achieved on the graphene surface, with feature sizes ranging from 5 to 25 nm. Nanoscale Fourier transform infrared spectroscopy (nano‐FTIR) revealed surface modifications induced by the nanopatterning. Furthermore, the effect of laser exposure time on the nanopattern morphology was explored. At shorter exposure times, nanoblister‐like structures formed with vertical feature height in the order of 1–2 nm, while longer exposure times resulted in the formation of distinct punch holes. Although laser‐aided technology offers several advantages, challenges remain in achieving sub‐10 nm resolution, controlling surface modifications, minimizing surface roughness, and material degradation.^[^ ^19^, ^20^, ^21^ ^]^ Ongoing efforts also focus on enhancing pattern flexibility, enabling operation under ambient conditions, preventing contamination, and chemical damage. In this work, we have successfully tackled all these challenges through our novel approach, offering significant advancements in 2D material nanopatterning techniques via two‐photon oxidation. This breakthrough enhances the tunability of graphene's electronic, optical, mechanical, and chemical properties and opens new possibilities for 2D material‐based device applications.

## 2 Results and Discussion

### 2.1 s‐SNOM Driven Optical Graphene Nanopatterning

**Figure 1:**
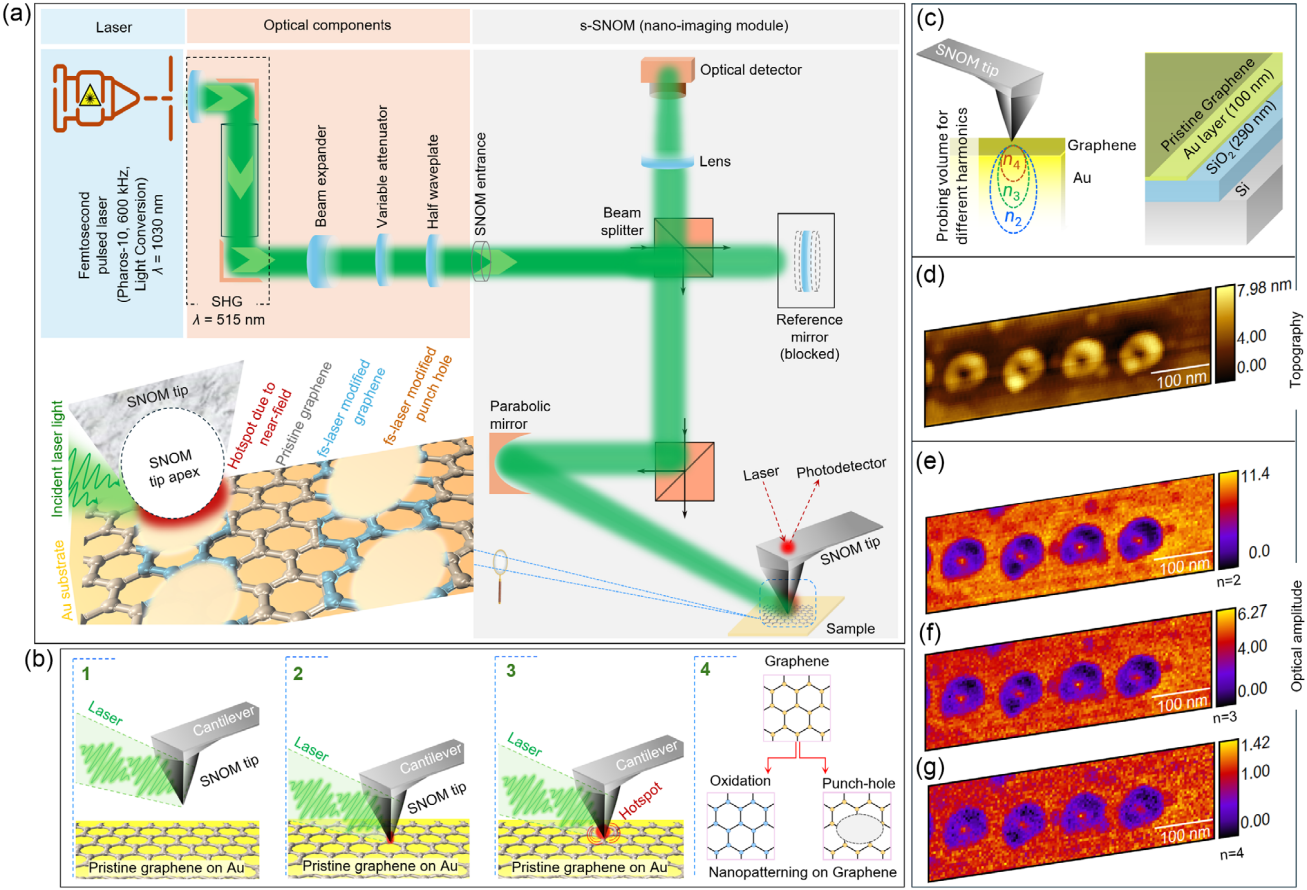
Schematic and procedure of graphene nanopatterning experiment. a) Schematic illustration of the experimental setup showing the graphene nanopatterning system, which includes a femtosecond laser as the light source, s‐SNOM, and integrated optical components for combining the laser with the s‐SNOM system. b) Detailed procedure for graphene nanopatterning, highlighting the key experimental steps necessary to achieve the desired nanopatterning on graphene. c) Schematic representation of the s‐SNOM imaging, illustrating the simultaneous measurement of multiple harmonics (n). The inset shows that the sample consists of graphene on a 100 nm‐thick gold layer, supported by a silicon substrate with a 290 nm oxide layer. d) A topographical image of the nanopatterned area was recorded using s‐SNOM, revealing the formation of a punch hole in the graphene. e–g) Broadband optical amplitude images of the nanopatterned area obtained with s‐SNOM at the 2nd, 3rd, and 4th harmonics, with the reflectivity scale showing the contrast between pristine and patterned graphene regions, indicating distinct optical responses of different harmonics. Here, the scale bar is 100 nm. Experimental parameters: laser power 0.7 mW and exposure time 120 sec.

**Figure** **1**a illustrates the experimental setup for graphene nanopatterning in ambient air. This setup comprises three primary components: femtosecond laser, s‐SNOM, and optical components (all instrumentation details are in the experimental section). Pulsed laser light from the femtosecond laser is guided into the s‐SNOM via optical components that increase the beam size and control the angle of linear excitation polarization. The sample, graphene deposited on a gold substrate, is placed on the sample stage of s‐SNOM for direct laser nanopatterning. The s‐SNOM microscopy image of the sample is shown in Figure S1a (details of graphene on gold fabrication can be found in the experimental section). Raman spectroscopy measurement, shown in Figure S1b, was performed to test the quality and structural integrity of the graphene. Figure S1b, Supporting Information shows two prominent peaks, the G peak at 1593 cm^−1^ and the 2D peak at 2708 cm^−1^. The G peak arises from the in‐plane vibrations of *sp* ^2^‐bonded carbon atoms and is a signature of the graphitic structure. The 2D peak, resulting from a second‐order two‐phonon scattering process, is sharp, symmetric, and intense, suggesting that the graphene is mostly a monolayer.^[^ ^45^ ^]^ Figure 1b illustrates the detailed experimental procedure for precise graphene nanopatterning (detailed experimental steps are given in the experimental section). The process begins by aligning the optical components to direct the laser light near the s‐SNOM tip. The laser light is coupled using a parabolic mirror integrated within the s‐SNOM, confirmed by a strong near‐field signal with a high signal‐to‐noise ratio across various harmonics. During alignment, the s‐SNOM operates in tapping mode to minimize tip‐sample interaction forces; however experiment was conducted in contact mode. The nanopatterning experiment was performed using a 0.7 mW laser power with an exposure time of 120 s.

s‐SNOM imaging was employed to investigate the infrared reflection properties of nanopatterned graphene. In this technique, the tip height is modulated at a specific frequency, and the detector signal is measured at higher harmonics.^[^ ^46^ ^]^ Higher harmonics are more sensitive to surface features, while lower harmonics probe deeper into the material (schematically illustrated in Figure 1c).^[^ ^47^ ^]^ This allows the separation of surface effects from bulk properties and enables nanoscale depth profiling. Figure 1d presents the topographical image of the nanopatterned graphene surface, captured using s‐SNOM, which reveals the surface morphology. Circular nanopunch hole patterns with surrounding elevated rings are visible, with the topography scale indicating height variations of up to ≈8 nm. Figures 1e–g provide s‐SNOM broadband (mid‐IR range) optical amplitude images, showcasing the nanoscale optical properties of the nanopatterned graphene. These images demonstrate a clear contrast between pristine graphene regions and punch‐hole patterned areas, highlighting differences in optical amplitude signals across multiple harmonics. The 2nd harmonic image shows the strongest contrast, with distinct low‐intensity regions concentrated at the edges of the punch holes. Although the optical amplitude decreases in the 3rd and 4th harmonics, the spatial distinction between patterned and unpatterned regions remains prominent. These variations across harmonics underscore the sensitivity of graphene's optical response to structural modifications, providing insights into scattering and localized light‐matter interactions within the nanopatterned areas.

**Figure 2:**
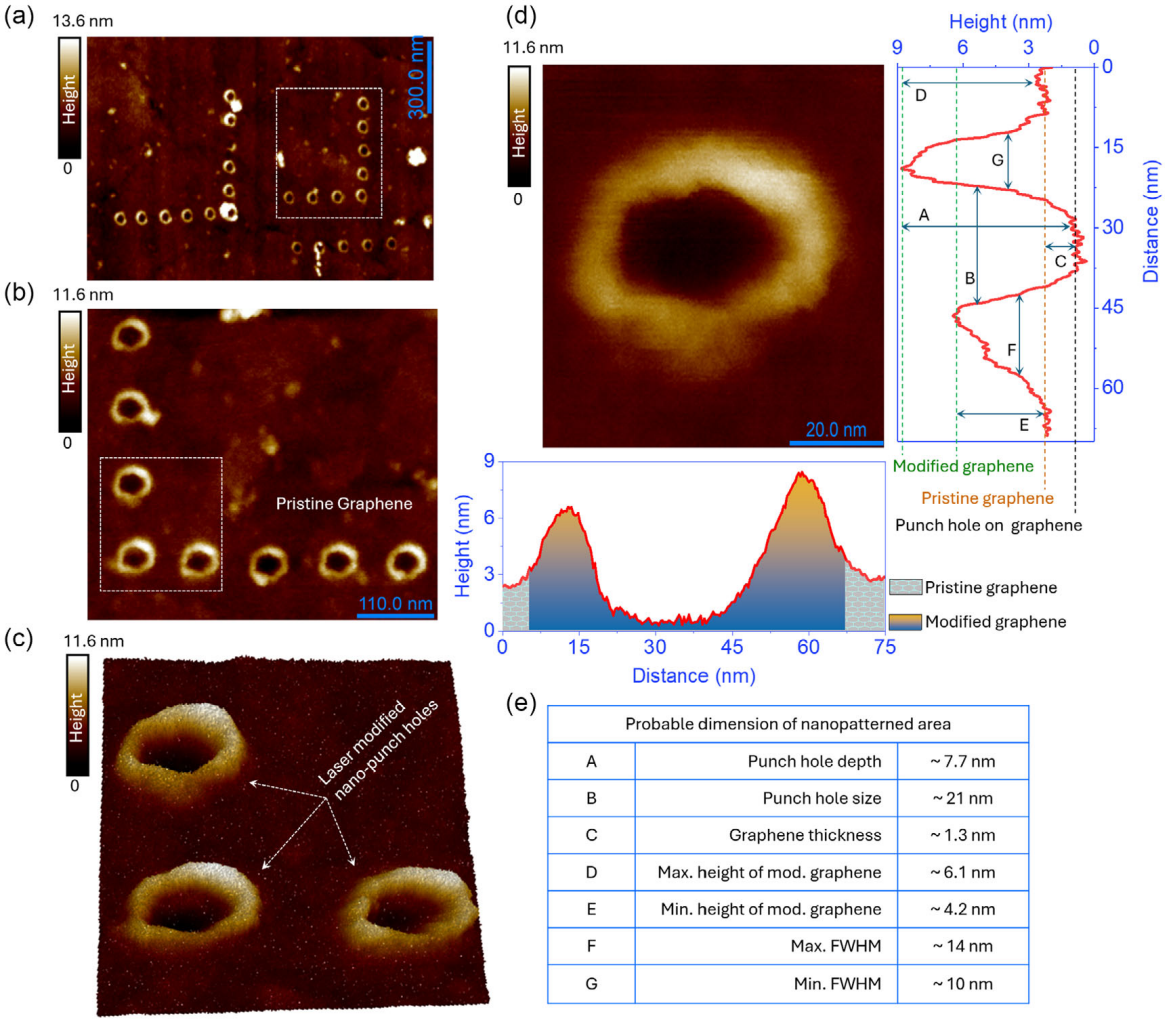
AFM topography images of nanopatterned graphene. a) AFM image shows an array of nanopunch holes formed on the graphene surface. Nanopunch holes were created using a laser power of 0.7 mW with an exposure time of 120 s. b) A 90°‐rotated and magnified view of the region marked by the white dotted box in (a), provides a closer look of the nanopatterned area. c) The AFM 3D topography of the magnified section in (b), illustrates the depth and morphology of the nanopunch holes. d) AFM image of a single nanopunch hole, accompanied by horizontal and vertical height profiles with the detail dimensions of a single nanopunch hole. e) Summary of the probable dimensions of the nanopatterned area depicted in (d) in tabular format.

The synergy between s‐SNOM and femtosecond lasers enables precise graphene nanopatterning by combining spatial confinement with ultrafast energy delivery. The system achieves precise control over the nanopatterning process by coupling the spatial resolution of s‐SNOM with the temporal precision of the femtosecond laser. The s‐SNOM tip confines the electromagnetic field to a nanoscale hotspot, focusing femtosecond laser energy into a region comparable to the dimensions of the tip apex. This localized near‐field enhances laser‐graphene interactions, allowing controllable super‐resolution nanopatterning. A crucial element of the nanopatterning experiment is the precise coupling of the femtosecond laser to the s‐SNOM tip, achieved by adjusting the parabolic mirror to focus the laser at the tip apex. Near‐field signals, sensitive to the mirror's position, provide real‐time feedback for fine‐tuning the alignment, ensuring the laser remains confined to the nanoscale region near the tip. This feedback mechanism is vital for achieving precise and reproducible graphene nanopatterning. Ultrashort pulses from the femtosecond laser deliver high‐precision energy with minimal heat diffusion, reducing collateral damage to adjacent areas. These pulses trigger nonlinear optical effects and localized electronic excitation in graphene, leading to multiphoton absorption and photoablation. This results in activating carbon‐carbon bonds, possibly photochemical functionalization/ oxidation, and creating well‐defined nanoscale patterns. As shown in the inset of Figure 1a, the interaction between the graphene surface and the laser‐coupled s‐SNOM tip creates a hotspot at the tip apex. In this technique, the localized interaction produced a single nanopunch hole on the graphene surface. Sub‐nm material removal was observed at the center of the punch hole (see below **Figure** **2**), while the periphery showed pristine graphene converting to modified graphene, potentially through oxidative functionalization.

### 2.2 Topographical Characterization of Nanopatterned Graphene

Figure 2 presents atomic force microscopy (AFM) images and analyses of nanopunch holes formed on a graphene surface, showcasing their morphology and dimensions. Figure 2a displays a wide‐area AFM image of the nanopatterned area, showing an array of circular punch holes, while Figure 2b provides a magnified view of the region marked in Figure 2a, clearly distinguishing between pristine graphene and laser‐modified regions. A prominent height contrast between pristine and nanopatterned graphene is evident. All experimental parameters were kept constant to ensure reproducibility, and the results confirm that the nanopatterning process is consistent. We replicated the patterning experiment on graphene at different locations using identical parameters. The corresponding AFM images are presented in Figure S1c and d. The 3D AFM topography in Figure 2c further emphasizes the depth and morphology of the nanopunch holes, revealing their symmetrical structure, which reflects a controlled and reproducible patterning process.

The statistical analysis of nanopunch hole dimensions, presented in Figure S2, shows the precision and reproducibility of our nanopatterning technique. The measured standard deviations (SD) for depth, horizontal (along x‐axis) size, and vertical (along y‐axis) size are 0.4, 1.46, and 1.14 nm, respectively. Both the horizontal and vertical dimensions exhibit low variability, with coefficients of variation (CV) of 2.69% and 3.06%, respectively, indicating excellent control over lateral feature sizes. The depth shows slightly higher variability (CV = 5.76%), which may be attributed to minor fluctuations in laser energy deposition or tip–sample interactions. Overall, the consistently low CV values (<6%) and SD across all parameters confirm the high precision and reliability of our nanopatterning method (For details, see section E of the Supporting Information).

Figure 2d provides a high‐resolution AFM image of a single nanopunch hole along with its height profile, offering a detailed cross‐sectional analysis of both vertical and horizontal variations across the graphene surface. The profile reveals three distinct regions: *pristine graphene*, forming the unmodified baseline with a consistent height of ≈1 nm, corresponding to the natural thickness of the graphene sheet; the *punch hole*, the central, deepest part of the profile, with a measured depth of ≈1 nm compared to the pristine graphene level and a width of ≈21 nm (comparable to the diameter of the s‐SNOM tip apex). The sharp edges and relatively flat bottom of the punch hole suggest precise material (presumably graphene) removal during laser processing. Alternatively, graphene could still be present, but the adhesion to the substrate could have changed. Surrounding the punch hole is the *modified graphene*, reflecting structural changes due to laser interactions, with heights ranging from ≈4.2 to ≈6.1 nm, and the full width at half‐maximum (FWHM), varies between ≈10 and ≈14 nm (Figure 2e).

Moreover, the tip apex quality before and after the experiment was analyzed using a scanning helium ion microscope. Figure S1e, Supporting Information shows the pristine, sharp tip‐apex before the experiment, while Figure S1f depicts the tip‐apex after nanopatterning. A magnified view in Figure S1g highlights deformation from prolonged AFM contact mode use, with the inset providing a detailed color‐rendered 3D view of the bent tip. Despite slight bending, the tip remained sharp, maintaining an apex size of ≈34 nm, close to its original condition even after 10 h of use. To evaluate the impact of tip degradation on patterning precision, we monitored changes in nanopunch hole dimensions over extended use. While the tip remained largely stable throughout most of the experiments (Figure S2), a gradual increase in feature size and asymmetry was observed in one instance (Figure S3), likely due to the slight bending of the tip apex (detailed analysis and supporting data are provided in section F of the Supporting Information).

### 2.3 Nano‐FTIR Characterization of Nanopatterned Graphene

**Figure 3:**
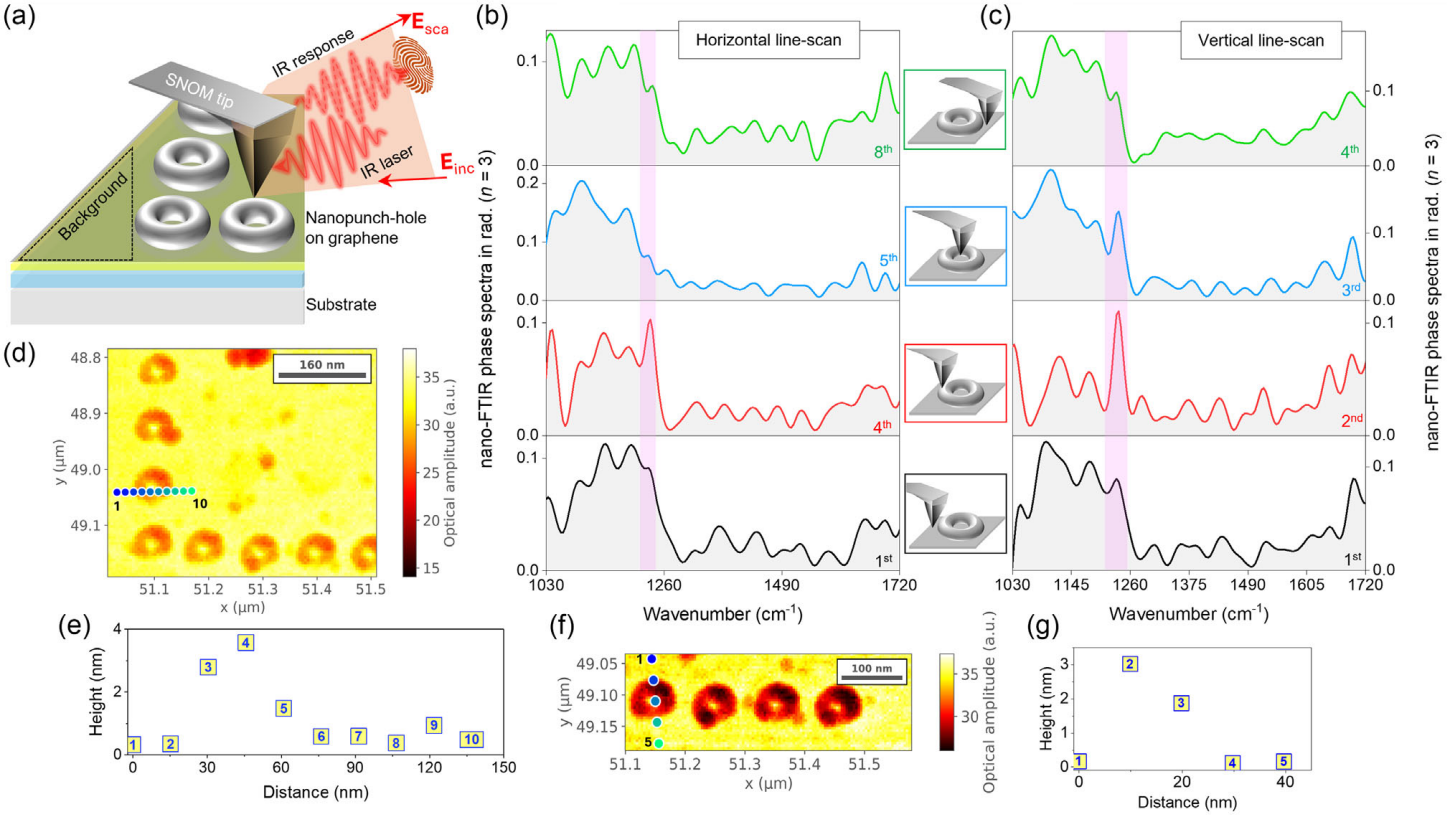
Nano‐FTIR characterization of nanopatterned graphene. a) Illustration of typical nano‐FTIR measurements over nanopunch holes, where reference spectra are collected from pristine graphene regions outside the nanopatterned area. b–c) Nano‐FTIR phase spectra of the horizontal and vertical scan for the third harmonic, respectively, demonstrating the spectral signatures across the nanopunch structures. Insets here reveal the tip location during the nano‐FTIR measurement. d) Optical broadband amplitude map showing horizontal line scan locations across nanopunch holes (indicated by circular dots). This line scan contains ten scan points, where 1 and 10 represent the 1st and last scan points in the line scan. e) The height profile corresponds to the horizontal line scan of ten points. f) Optical broadband amplitude map of nanopunch holes showing the locations of vertical line scan measurements, marked by circular color dots. Five scan points are analyzed, where 1 and 5 represent the 1st and last scan points in the line scan. g) The height profile corresponds to the vertical line scan of five points.

We used the nano‐FTIR technique to characterize the nanopatterned area of graphene, as shown in **Figure** **3**. Figure 3a presents a schematic illustration of the nano‐FTIR technique; pristine graphene regions near the patterned area serve as the background reference for the spectral measurements. Detailed instrumental information about nano‐FTIR is given in the experimental section, and the theory of nano‐FTIR of molecular vibrations is provided in section A of the Supporting Information. In brief, nano‐FTIR combines s‐SNOM with a broadband infrared source to obtain local spectral information. It performs FTIR spectroscopy using the backscattered light from the s‐SNOM tip. An interferogram from the scattered light is recorded by linearly moving the reference mirror of a Michelson interferometer and applying higher harmonic demodulation to suppress background noise. The Fourier transformation of the interferogram provides the near‐field amplitude and phase spectrum of the sample.^[^ ^48^, ^49^, ^50^ ^]^ The near‐field signal can be analyzed using the approach curves and localization lengths (*Z*_*loc,n*_) derived from the approach curve,^[^ ^51^, ^52^ ^]^ which provides valuable insights into the near‐field interactions in both pristine and nanopatterned graphene regions (section I of the Supporting Information and Figure S4). Nanopatterned regions show consistently shorter localization lengths than pristine graphene, indicating stronger near‐field coupling due to defects and functionalized edges.^[^ ^44^, ^53^ ^]^ Higher harmonics (*n* = 2,3,4) exhibit faster signal decay and smaller localization lengths, reflecting enhanced spatial confinement and sensitivity to nanoscale variations.

Figure 3f illustrates the locations of the vertical line scan, as shown in the broadband optical image. This scan consists of five points, with their corresponding baseline‐corrected height profiles presented in Figure 3g. The 1st point is located on pristine graphene, while the 2nd point shows an increase in height compared to the 1st, indicating that the measurement was taken from the nanopunch hole area. This aligns with the understanding that the nanopatterned regions exhibit increased height compared to pristine graphene. In contrast, the 4th and 5th scan points were collected outside the nanopunch hole, as supported by the height profile (Figure 3g). The corresponding third harmonic background‐corrected phase spectra are shown in Figure 3c. The nanopunch holes were further characterized using a horizontal nano‐FTIR line scan. The scan locations are displayed in the optical amplitude image in Figure 3d, with the corresponding height profile in Figure 3e. The horizontal line scan consists of ten points (all the spectra are given in Supporting Information as Figure S5), similar to the vertical line scan, the 1st and last scan points are represented as 1 and 10. The height profile reveals that the 4th scan point has the maximum height, indicating that this measurement was taken from the elevated region of the nanopunch hole, while the 5th scan point corresponds to the center of the nanopunch hole, where the height is lower. The recorded third harmonic phase spectra are shown in Figure 3b, and the insets between Figure 3b,c indicate the tip's location during spectral measurements.

Although, the phase spectra in Figure 3b,c represent line scans in different directions, they can still be correlated. In both cases, the black spectra (1st scan point in both vertical and horizontal scans) correspond to measurements taken on pristine graphene or outside the nanopunch hole. The red spectra (2nd in the vertical and 4th in the horizontal scan) represent data recorded at the maximum height, as confirmed by the height profile, indicating measurements from the elevated regions of the nanopunch hole. The blue spectra (3rd in the vertical and 5th in the horizontal scan) were recorded from the center (or close to the center) of the nanopunch hole, where the height is lower than the maximum point, as verified by the height profile. Finally, the green spectra (4th in the vertical and 8th in the horizontal scan) correspond to regions outside the nanopunch hole, where the height is comparable to the first scan point, confirming their location on pristine graphene. The third harmonic phase spectra shown in Figure 3b,c reveal spectral differences between the nanopatterned and pristine graphene areas. Specifically, the peak at 1236 cm^−^^1^, likely associated with C–O–C epoxy vibrations,^[^ ^54^ ^]^ appears strong in the red spectrum (1236 cm^−1^ region from Figure 3b,c plotted separately in Figure S5a and b to clearly show the difference in nano‐FTIR phase values of pristine graphene and nanopatterned graphene region). In contrast, this absorbance band is much weaker in the black and green spectra, which correspond to pristine graphene regions. This functional group is more prominent in the nanopatterned areas, likely due to the introduction of oxygen‐containing groups during the nanopatterning process. In pristine graphene, these functional groups are naturally absent or much less abundant due to the material's intrinsic *sp* ^2^‐hybridized structure and low defect density. Additionally, it is observed that in the blue spectrum, the 1236 cm^−1^ peak is strong for vertical line scans, whereas weak for horizontal scans. The absence of the peak in horizontal scans can be explained by the optical amplitude image, where the color contrast between the center of the nanopunch hole and the surrounding pristine graphene is almost identical. However, in the vertical scan, the tip may not be positioned exactly at the center of the nanopunch hole but rather on its surface, close to the center, which could account for the observed spectral differences. Notably, the findings from the nano‐FTIR characterizations align with our previous work,^[^ ^40^ ^]^ in which we investigated the chemical composition via micrometer‐scale X‐ray photoelectron spectroscopy (XPS) of diffraction‐limited, femtosecond pulsed driven two‐photon oxidized single‐layer graphene under an air atmosphere. XPS data demonstrated that the highly oxidized regions were majorly dominated by hydroxyl (C–OH) and epoxide (C–O–C) groups.

Therefore, the nano‐FTIR characterizations suggest that the nanopunch holes are regions of enhanced chemical activity or structural modification, possibly due to localized near‐field, defects, or increased exposure to oxygen‐containing groups during the fabrication process, which clearly refers to two‐photon oxidation of graphene.^[^ ^26^ ^]^ To estimate the optical conditions enabling two‐photon oxidation, we calculated a far‐field peak intensity of ≈2.97 × 10^1^ ^1^ W/cm^2^ and a near‐field intensity at the tip apex of ≈3.8 × 10^1^ ^2^ W/cm^2^ (detailed conceptual calculations are given in section G, Supporting Information), based on tip geometry and electromagnetic simulations. These values lie within the established range for two‐photon oxidation of graphene, as reported in prior studies.^[^ ^26^ ^]^ In laser‐based nanopatterning, it is critical to assess whether thermal effects contribute to the observed surface modifications. However, due to the long inter‐pulse interval of 1.67 μs and the significantly shorter thermal diffusion times in graphene (≤65 ps), localized heating from each femtosecond pulse fully dissipates before the arrival of the next pulse. This effectively rules out photothermal accumulation, supporting two‐photon photochemical oxidation as the dominant mechanism (see section H, in Supporting Information for detailed explanation).

### 2.4 Effect of Laser Exposure Times on the Graphene Nanopatterning

**Figure 4:**
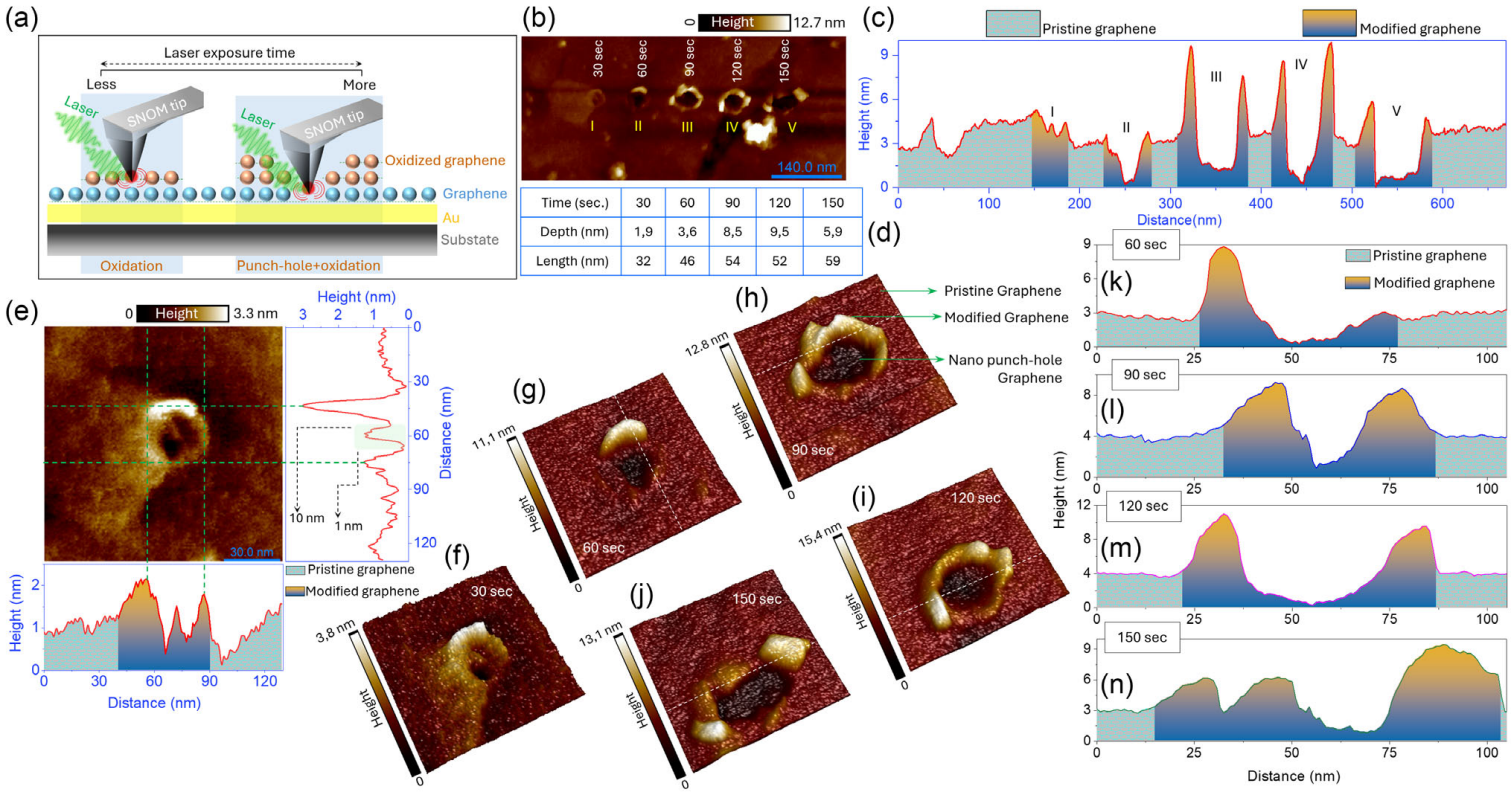
Influence of laser exposure time on the graphene nanopatterning. a) Schematic representation illustrating the effect of laser exposure time on the graphene surface, where shorter exposure times lead to only oxidation and longer times result in nanopunch holes with sharp oxidized boundaries, due to increased interaction with the optical near‐field at the SNOM tip apex. b) AFM image of nanopatterned graphene, showing features formed by varying laser exposure times. c) Height profiles of the patterned regions extracted from (b), show the progressive changes in depth and lateral dimensions with increased exposure time. d) Quantitative summary of the depth and length of the features as a function of laser exposure time. e) Magnified AFM image of the nanoblister formed at 30 sec exposure, along with its horizontal and vertical height profiles, showing a height of 1–2 nm and a lateral size of ≈10 nm. f) 3D AFM representation of the nanoblister, showcasing its smooth and rounded morphology. g–j) 3D AFM images of patterned features created at 60, 90, 120, and 150 sec exposure times, demonstrating the transition from nanoblisters to nanopunch holes with increasing depth and sharper edges. The height profiles corresponding to these 3D images, taken along the white dashed lines, are shown in (k–n), emphasizing the progressive increase in depth (up to ≈5.9 nm) and lateral size (up to ≈59 nm) at 150 sec exposure. Experimental parameters: laser power 0.7 mW and exposure time 30 sec to 150 sec.

**Figure** **4** illustrates the impact of varying laser exposure times on the graphene nanopatterning process. As previously discussed, this process is governed by the optical near‐field effect at the SNOM tip apex, where highly concentrated energy within a nanoscale region induces photochemical oxidation and/or ablation of graphene, progressively modifying its structure. The exposure time plays a critical role in controlling the degree of this interaction, thereby shaping the morphology of the patterned features. The schematic in Figure 4a provides an overview of this mechanism, contrasting the effects of short and long exposure times. Figure 4b visually demonstrates this transformation through AFM images, revealing a progression from nanoblister formation (e.g., 30 s) to nanopunch hole creation (e.g., 90 s). Figure 4c presents horizontal height profiles, illustrating the gradual increase in both depth and lateral size, while Figure 4d quantifies these changes. At 30 s, the localized interaction results in a nanoblister with a height of ≈1–2 nm and a lateral size of ≈10 nm, as shown in Figure 4e. This feature represents the early stages of oxidation, where the graphene undergoes partial modification, leading to the formation of a nanoblister. The corresponding 3D AFM image in Figure 4f further confirms the smooth, rounded morphology characteristic of such nanoblisters. Additionally, the AFM image indicates that nanoblisters form on the top of the graphene double‐layer region. The observed vertically modified dimension of 1–2 nm (Figure 4e) for nanoblisters is not an artifact of graphene's inherent thickness but rather a direct result of the controlled interaction between the femtosecond laser and the graphene surface.

As the exposure time increases (60–150 s), the features evolve into deeper structures with sharper edges, marking the transition to nanopunch holes. This transformation is evident in the 3D AFM images (Figure 4g–j) and their respective height profiles (Figure 4k–n), where the depth reaches ≈5.9 nm, and the lateral size expands to ≈59 nm at 150 s. These 3D AFM images clearly reveal the breaking of graphene and its subsequent rolling into elevated structures, a phenomenon particularly noticeable in Figure 4j. Unlike the smooth ring structures observed in Figure 2, Figure 4j distinctly shows torn graphene, with no continuous ring but rather fragmented, lifted edges, confirming that the graphene is broken and has rolled off the substrate. This suggests the graphene remains whole at low laser exposure time (Figure 2c), whereas it is completely broken (Figure 4j) at higher exposure time.^[^ ^55^ ^]^ This random tearing process naturally produces asymmetrical structures. As long as graphene stays unbroken, the structures are symmetrical and smooth. As soon as tearing and ablation occur, the structures become asymmetrical, and the control over them diminishes.

The localized energy transfer mechanism underlying this variation can be summarized as follows: when the laser is coupled with the SNOM tip, an intense, localized hotspot forms at the tip apex due to the optical near‐field effect. This hotspot interacts strongly with the graphene surface beneath it. For shorter exposure times, the interaction is brief, resulting in minimal oxidation and the formation of a small, elevated nanoblister. With longer exposure times, the field has more time to interact with the surface, leading to deeper oxidation and the creation of nanopunch holes. Graphene oxidation, as known, involves the disruption of its *sp* ^2^‐bonded carbon network and the incorporation of oxygen functional groups. This process is energy‐dependent, with shorter exposures causing partial oxidation, while prolonged exposures trigger significant chemical and structural modifications. By precisely controlling the laser exposure time, the depth and lateral dimensions of the patterned regions can be tuned, showcasing the versatility and tunability of the proposed nanopatterning technique.

In this study, we define processing resolution as the smallest reproducible and controllable structural modification, both vertically and laterally, that our technique can induce on pristine graphene. We demonstrate sub‐5 nm vertical modulation capability and achieve lateral feature sizes ranging from 10 to 50 nm, as confirmed by AFM measurements. Furthermore, we have compared various near‐field driven nanopatterning approaches that integrate tip as probes and lasers (Table 2, Supporting Information). The smallest reported feature size in the literature is ≈5 nm. To the best of our knowledge, this is the first reported near‐field‐driven nanopatterning technique achieving a sub‐5 nm minimum feature size. From the conceptual standpoint, the primary limitations of the current experiment stem from the dimension of the tip apex. However, sub‐nm lateral dimension can be achieved by utilizing an atomically confined near‐field active tip.^[^ ^56^ ^]^ While this study is currently at the proof‐of‐principle stage, we propose several strategies to address throughput limitations. Automating the scanning and patterning process, along with optimizing laser parameters, could significantly reduce processing times. Additionally, utilizing a laser with a higher repetition rate would enhance patterning efficiency and scalability for larger‐area applications. For example, by using the femtosecond oscillator, the patterning speed could be potentially improved by a factor of 100. In addition to graphene on gold, we also conducted preliminary nanopatterning experiments on graphene supported by SiO_2_/Si substrates. However, due to weaker near‐field enhancement and potential polymer residues on the dielectric‐supported graphene, the resulting patterns were less systematic/well‐defined (AFM image of nanopatterned graphene on SiO_2_/Si substrate is given in Figure S6, Supporting Information). Given the technological relevance of dielectric substrates in nanoelectronics and the insights gained from our current work, we plan to revisit and optimize patterning on graphene/dielectric systems in future studies, with improved sample preparation and refined laser parameters.

## 3 Conclusions

In summary, this study establishes a novel approach for graphene nanopatterning using a femtosecond laser coupled with s‐SNOM in ambient conditions. The combination of spatial resolution of s‐SNOM with femtosecond laser excitation allowed localized light‐matter interactions, minimizing collateral damage, and super‐resolution nanopatterning with reproducible nanoscale 2D materials modifications. s‐SNOM imaging revealed punch‐hole patterns on the graphene surface with distinct topographical and optical contrasts, emphasizing differences in reflectivity and surface morphology. AFM analysis of graphene nanopatterning revealed the precise formation of circular nanopunch holes with consistent morphology and dimensions. The replication of nanopatterning across different areas confirmed the reproducibility of the experiment. Despite prolonged AFM contact mode nanopatterning, the s‐SNOM tip retained its functionality and sharpness, highlighting the durability of the system. The nano‐FTIR technique provides detailed characterization of nanopatterned graphene, revealing distinct chemical and structural modifications compared to pristine regions. Nanopunch holes exhibit enhanced near‐field coupling, shorter localization lengths, and higher vibrational intensities for oxygen‐related functional groups, such as C–O–C. The study supports that the nanopatterning process introduces oxygen‐containing groups at defect sites, showing increased chemical activity in these areas. Varying laser exposure times demonstrate precise control over the morphology of the nanopatterned features, transitioning from nanoblisters at shorter exposures to well‐defined nanopunch holes at longer exposures. Hence, this work demonstrates the potential of the femtosecond laser coupled s‐SNOM platform as a reproducible and damage‐free approach for graphene nanostructuring/nanoscale functionalization. This optical near‐field driven nanopatterning sets a new benchmark for direct laser writing on 2D materials by achieving sub‐5 nm vertical control and sub‐10 nm lateral modification under ambient conditions, and opening avenues for high‐resolution nanofabrication, advanced 2D materials‐based device architecture, sensing technologies, quantum photonic, and optoelectronic devices, where precise material modification is important.

## 4 Experimental Section

4.1

4.1.1

##### Experimental and Characterization Details

Fabrication of “graphene on gold” sample: The graphene‐on‐gold sample was fabricated by first growing graphene on a thin copper film (400 nm) that was evaporated onto a 5 × 5 mm piece of sapphire wafer. The copper surface was annealed at 1040 °C for 15 min in an atmosphere of argon (95%) and hydrogen (5%) inside a tube furnace. Following annealing, the graphene film was grown by heating the furnace to 1090 °C and introducing a methane (1%)/argon (99%) gas mixture into the furnace chamber. The growth process lasted 20 min, after which the samples were removed from the furnace. To deposit the gold layer, 100 nm of gold was evaporated onto the graphene samples using an electron beam (e‐beam) evaporation system. This system operates by heating a crucible containing the target metal with electron beam bombardment, causing the metal to vaporize. The vaporized metal molecules then condense onto the sample surface, forming a thin film. The evaporation was conducted under a high vacuum (≈10^−9^ mbar) to minimize contamination, with an evaporation rate of 0.5–0.8 Å/s. At this stage, the samples consisted of a sapphire/copper/graphene/gold stack. The copper layer was removed using a wet etching process to isolate the graphene and gold layers. To ensure the gold layer was properly detached from the sapphire surface, the edges of the gold film were carefully cut with a scalpel, as material deposition during evaporation might have adhered to the edges of the chip. The chip was then placed at the bottom of a glass container, and a few milliliters of iron chloride (FeCl_3_) etchant were added around the sample, ensuring the edges touched the liquid while keeping the chip submerged but stationary. Over 4–6 h, the etchant gradually removed the copper layer.

Once the etching was complete, the sample was carefully transferred into a container filled with deionized (DI) water. At this point, the graphene/gold stack floated on the water surface, while the sapphire piece was discarded. The floating film was sequentially transferred through multiple cups of DI water and one cup containing 12% hydrochloric acid (HCl). The water baths removed residual etchant, and the HCl bath ensured the complete removal of any copper residues. After the final water bath, the film was flipped to position the graphene side facing upward. This flipping step involved scooping up the film with a piece of bare silicon, flipping it on the water surface, and placing it back upside down. While challenging, this step was necessary to expose the graphene layer for subsequent use. Finally, the graphene/gold stack was transferred to the target substrate, a 5 × 5 mm SiO_2_ chip (with a 290 nm oxide layer) prepatterned with a palladium (25 nm) metal grid for measurement alignment. The sample was then gently blow‐dried using a nitrogen gun and stored in a vacuum chamber for 24 h.

*Raman spectroscopy*: The Raman spectra were recorded with a commercial Raman microscope (Nicolet DXR) from ThermoFischer, equipped with a 50x objective lens and continuous wave (CW) laser. The excitation wavelength was 532 nm, 900 lines/mm gratings, and the laser power was 1 mW.

*AFM imaging*: The sample was imaged using an AFM (Bruker, Dimension Icon) with peak force tapping mode. During imaging, we used ScanAsyst Air probes from Bruker with the peak force limited to 2.5 nN. AFM images were processed using NanoScope Analysis 1.9 software.

*Coupling s‐SNOM and femtosecond laser*: Femtosecond laser (Pharos‐10, 600 kHz, Light Conversion Ltd, Lithuania) was used as the light source in the laser writing experiment. The output from the SHG was 515 nm, with a pulse duration of ≈250 fs. The femtosecond laser beam was expanded to 10 mm in diameter prior to the coupling into the input port of the SNOM instrument. The polarization orientation of the laser beam was adjusted to give the best near‐field signal by using a half‐wave plate on the rotational mount. Linear polarization was used for 515 nm excitation. The direction of nominally s‐polarized light was slightly adjusted to optimize the near‐field signal. Pt–Ir‐coated Si tip (ARROW‐NCPt, Nanoworld) was used for nanopatterning, cantilever resonance tapping frequency 276 kHz, amplitude 473 nm, Q‐factor: 580.3, femtosecond laser power of 0.7 mW and contact force: 20.626 nN.

*s‐SNOM imaging and nanoFTIR*: NeaSCOPE instrument (Attocube systems AB) equipped with a tunable broadband femtosecond mid‐IR laser source operating at 650–2170 cm^−1^ range (Toptica Photonics AG) was used for broadband s‐SNOM imaging and nanoFTIR measurements. NanoFTIR spectra were collected with a spectral resolution of 16.66 cm^−1^ using 20 ms for pixel time and 10 averages. Pt/Ir‐coated spectroscopy tips (attocube systems AB, nominal Ω = 285 kHz) were used in broadband imaging and spectroscopy. To counter the mechanical drift of the AFM stage, the tip was relocated between each spectra acquisition by scanning a new topography image. All the nano‐FTIR data were processed by using commercial neaPLOT and Originpro 2017 software.

##### Experimental Graphene Nanopatterning Procedure

1) The initial step involves directing the laser light close to the s‐SNOM tip. This was achieved by adjusting the alignment of the optical components, and precise control of these alignments ensures that the laser light is effectively guided to the desired location. 2) Once the laser light was in proximity to the s‐SNOM tip, the next step was to couple the light to the tip. This was facilitated by a parabolic mirror integrated within the s‐SNOM. The coupling was confirmed by observing the near‐field signal generated by the laser interacting with the tip (also tip must be close to the surface). 3) To optimize the tip‐laser interaction, the x‐y‐z position of the parabolic mirror is finely tuned. A strong near‐field signal and a high signal‐to‐noise ratio across various harmonics confirm the ultimate laser coupling with the tip. At this stage, the s‐SNOM tip must be retracted, and the optical path between the laser and s‐SNOM was temporarily blocked. After completing the alignment, the system was set up for nanopatterning. The optical alignment process was performed while the s‐SNOM operates in tapping mode to minimize tip‐sample interaction forces. Once a specific region of interest on the graphene surface was selected, the system was switched to contact mode. In this mode, the s‐SNOM tip was brought into close proximity to the graphene surface by approaching the tip, and the laser was unblocked to initiate the nanopatterning process. 4) Laser precisely irradiates the graphene surface while maintaining controlled tip positioning during the nanopatterning experiment. The Laser power of 0.7 mW with an exposure time of 120 s was used as the experimental parameter. Once the patterning was complete, the laser light was blocked, and the s‐SNOM tip was retracted to prevent any further interaction. Finally, s‐SNOM imaging was conducted to assess the quality of the nanopatterned graphene surface.

##### Statistical Analysis

All statistical findings are reported as mean ± SD. For example, the depth, vertical size, and horizontal size of punch holes were analyzed with CV values of 5.76%, 2.69%, and 3.06%, respectively. The sample size (N) for each analysis was specified in the corresponding figure legends (e.g., N = 10 for punch hole dimensions). All statistical analysis were performed using OriginPro software.

## Conflict of Interest

The authors declare no conflict of interest.

## Author Contributions

**Gour Mohan Das**: investigation; methodology; data curation; formal analysis; writing—original draft; writing—review and editing. **Eero Hulkko**: investigation; methodology; data curation; formal analysis; writing—review and editing. **Pasi Myllyperkiö**: conceptualization; investigation; methodology; formal analysis; writing—review and editing. **Andreas Johansson**: methodology; supervision; writing—review and editing. **Mika Pettersson**: conceptualization; resources; project administration; funding acquisition; supervision; writing—review and editing. All authors read and approved the final manuscript.

## Supporting information

Supplementary Material

## Data Availability

The data that support the findings of this study are available from the corresponding author upon reasonable request.
