# Supplementary material for: Near‐Field Optical Nanopatterning of Graphene
Source: Small Sci. 2025 Jun 30;5(8):2500184. doi: 10.1002/smsc.202500184 (PMC12362822; doi:10.1002/smsc.202500184)
Supplement: Supplementary file 1 — Supplementary Material [file SMSC-5-2500184-s001.pdf]

“Supporting Information” of

Near-Field Optical Nanopatterning of Graphene

Gour Mohan Das[1], Eero Hulkko[1,2], Pasi Myllyperkiö[1], Andreas Johansson[1,3] and Mika Pettersson[1]*

[1]*Nanoscience Center, Department of Chemistry, University of Jyväskylä, Jyväskylä, P. O. Box 35, FI-40014, Finland*

[2]*Nanoscience Center, Department of Biological and Environmental Science, University of Jyväskylä, Jyväskylä, P. O. Box 35, FI-40014, Finland*

[3]*Nanoscience Center, Department of Physics, University of Jyväskylä, Jyväskylä, P. O. Box 35, FI-40014, Finland*

*Corresponding author: mika.j.pettersson@jyu.fi

Contents:

A. Theory of nano-FTIR of molecular vibrations

B. Table 1: Comparison between EBL vs RIE for patterning of 2D materials

C. Table 2: Comparison between different near-field nanopatterning methods

D. Figure S1

E. Statistical quantification of nano-punch holes: Figure S2 and explanation

F. Impact of tip degradation on patterning precision: Figure S3 and explanation

G. Estimation of laser Intensity and near-field enhancement for two-photon oxidation

H. Clarification of thermal vs. non-thermal effects on nanopatterning

I. Details of the approach curve: Figure S4 and explanation

J. Figure S5

K. Nanopatterning of graphene on a SiO_2/Si substrate (Figure S6)

References

A. Theory of nano-FTIR of molecular vibrations

Nano-FTIR spectroscopy [1–4] for molecular vibrations is based on the interaction between the electromagnetic field of IR light and the localized near-field region at the apex of an oscillating metallic tip. The backscattered light is analyzed using an asymmetric Fourier transform spectrometer, which operates on the principle of a Michelson interferometer. In this setup, the sample and AFM tip are positioned within one of the interferometer arms. This setup allows the extraction of both amplitude $s(\omega)$ and phase $\phi(\omega)$ spectra of the scattered light. The complex scattering coefficient$\sigma(\omega) = s(\omega)e^{i\phi(\omega)}$ relates the scattered field $E_{sca}(\omega)$ to the incident field $E_{inc}(\omega)$ via $E_{sca}(\omega) = \sigma(\omega)E_{inc}(\omega)$. Background contributions are suppressed by demodulating the detector signal at a higher harmonic $n\Omega$ of the tip oscillation frequency Ω, and Fourier transformation of the demodulated interferogram yields the near-field spectrum $E_n(\omega) = \sigma_n(\omega)R(\omega)E_{inc}(\omega)$. Here, $R(\omega)$ represents the spectral response of the instrument, including the transmission properties, atmospheric absorption, and detector responsivity. Normalizing $E_n(\omega)$ with a reference spectrum $E_{n,ref}(\omega)$ obtained using a spectrally flat sample like Si, isolates the scattering coefficient $\sigma_n(\omega) \propto E_n(\omega)/E_{n,ref}(\omega)$, which encodes the tip-sample near-field interaction.

The scattering coefficient $\sigma_n(\omega)$ depends on the local dielectric function $\epsilon(\omega)$ of the sample, encapsulated in the surface response function $\beta(\omega) = \frac{\epsilon(\omega)-1}{\epsilon(\omega)+1}$. Using a point-dipole /finite-dipole model, the scattering coefficient is expressed as $\sigma(\omega, H) \propto \alpha_{eff}(f \cdot \beta)(1 + r_s)^2$, where α_{eff} is the effective tip polarizability, $f(H)$ is a tip-sample distance-dependent function, and r_s is the far-field reflection coefficient. For thin films and weak molecular oscillators, the scattering coefficient simplifies under a Taylor expansion, resulting in $\sigma_n(\omega) \propto \beta(\omega)$ after normalization. The imaginary part of $\beta(\omega)$, $Im\beta(\omega)$, correlates with the imaginary part of the dielectric function and the absorption coefficient $\kappa(\omega)$, directly linking the nano-FTIR absorption $a_n(\omega) \propto Im[\sigma_n(\omega)]$ with far-field absorption spectra $A(\omega)$. This model-independent relationship, $a_n(\omega) \propto A(\omega)$, explains the observed correspondence between near-field and far-field absorption, providing a robust framework for chemical identification and nanoscale spectroscopic analysis.

Definition of absorption and normalization of nano-FTIR spectra: nano-FTIR absorption, $a_n(\omega) = Im[\eta_n(\omega)] = Im\left[\frac{\sigma_n(\omega)}{\sigma_{n,ref}(\omega)}\right]$, the imaginary part of the normalized near-field spectrum $\eta_n(\omega)$. The imaginary part can be easily calculated from the amplitude and phase spectra, $Im[\eta_n(\omega)] = \frac{s_n(\omega)}{s_{n,ref}(\omega)} sin\left(\phi_n(\omega) - \phi_{n,ref}(\omega)\right)$. Here, $s(\omega)$ and $\phi(\omega)$ being the amplitude and phase of the near-field.

Normalized amplitude spectrum: $\frac{s_n(\omega)}{s_{n,ref}(\omega)}$ and normalized phase spectrum: $\phi_n(\omega) - \phi_{n,ref}(\omega)$

B. Table 1: Comparison between EBL vs RIE for patterning of 2D materials

Electron beam lithography (EBL) and reactive ion etching (RIE) are widely used techniques in nanofabrication, especially for creating patterns on 2D materials like graphene, MoS_2, etc. Below is a concise comparison table of these two techniques based on their features (pros and cons) in nanometer-scale patterning of 2D materials.

Parameters	Electron Beam Lithography (EBL)	Reactive Ion Etching (RIE)
Parameter	Uses a focused beam of electrons to directly write patterns onto a resist layer, which is later transferred to the material.	Uses chemically reactive plasma to selectively etch away material not protected by a mask or resist.
Resolution	Extremely high resolution (down to sub-10 nm).	Moderate resolution (typically 10–100 nm), depending on plasma chemistry and process conditions.
Precision	Excellent precision due to direct electron beam control.	Less precise than EBL, as it depends on the quality of the mask and uniformity of plasma etching.
Material Compatibility	Suitable for patterning 2D materials like graphene, MoS_2, etc., with minimal damage if optimized conditions are used.	Can cause some damage to 2D materials due to ion bombardment, but can be mitigated with low-energy plasma processes.
Throughput	Low throughput; the serial writing process makes it slow for large-area patterning.	High throughput; parallel processing allows faster fabrication over larger areas.
Cost	Expensive equipment and operation costs due to high-vacuum requirements and electron beam systems.	Relatively lower in cost compared to EBL, although it still requires specialized equipment for plasma generation.
Flexibility	Highly flexible; allows arbitrary pattern designs without the need for physical masks.	Limited flexibility; requires predefined masks or resists for selective etching.
Damage to 2D Materials	Minimal damage if optimized (e.g., low-energy electrons, careful resist development).	Potential for ion-induced defects and surface roughness, especially in sensitive 2D materials.
Applications	Ideal for prototyping, research, and small-scale production requiring high precision.	Best suited for mass production and large-scale patterning, where moderate resolution is acceptable.
Reference	Clericò, V., Amado, M., & Diez, E. (2020). Electron beam lithography and its use on 2D materials. In Nanofabrication: Nanolithography techniques and their applications (pp. 3-1). Bristol, UK: IOP Publishing.	He, T., Wang, Z., Zhong, F., Fang, H., Wang, P., & Hu, W. (2019). Etching techniques in 2D materials. Advanced Materials Technologies, 4(8), 1900064.

C. Table 2: Comparison between different near-field nanopatterning methods

Sl. No.	Nanopatterning Method	Laser (nm)	Near-field probe type	Substrate	Resolution (nm)	Ref.
1	Nanosecond laser irradiation of a STM tip	Nano and femtosecond laser	Scanning tunneling microscope	Gold film	20–30 nm	[5], [6]
2	FOLANT (Focusing of laser radiation in the near-field of a tip)	Nd:YAG laser (532 nm)	Scanning probe microscope	Gold on palladium	lateral resolution of around 10 nm	[7], [8]
3	Near-field femtosecond laser-induced nanofabrication	Femtosecond laser (800 nm)	Commercial atomic force microscopy	In, Au, Cu, FeCr on Si substrate	20–30 nm	[9]
4	Laser scanning probe microscope-based nano-processing	nanosecond pulsed laser	Atomic force microscopy	Gold film on Si	Depth around 15 nm	[10]
5	Tip enhanced near-field laser ablation	nanosecond pulsed laser (266 nm)	Atomic force microscopy	Gold, Tantalum and, Silicon	lateral resolution of 100 nm	[11]
6	Pulsed laser assisted nanopatterning combined with AFM	nanosecond pulsed laser (532 nm)	Atomic force microscopy	Copper film	10 nm resolution	[12]
7	Near-field two-photon nanolithography	Femtosecond laser (790 nm)	Contact mode commercial AFM	SU-8 photoresist (polymer)	70 nm resolution	[13]
8	Floating Tip Nanolithography	Femtosecond laser	Atomic force microscopy	AZ4620 photoresist film (polymer)	spatial resolution of ~20 nm	[14]
9	Apertureless near-field nanomachining	Femtosecond laser (800 nm)	Scanning probe microscopy	Thin gold films	10 nm spatial resolution	[15]
10	tip enhancement effect induced by nanosecond pulsed laser irradiation	Nanosecond pulsed laser (532 nm)	Atomic force microscopy	50 nm thick Au and Ag films	minimum lateral feature size around 16.65 nm	[16]
11	near-field scanning optical microscope combined with femtosecond laser	Femtosecond laser (400 nm)	Near-field scanning optical microscopy (NSOM)	Thin photo-resist film	feature width of 20 ± 5 nm	[17]
12	Nanopatterning using NSOM	Femtosecond laser (800 nm)	Near-field scanning optical microscopy (NSOM)	photoresist	width of 59 nm and as small as 24 nm	[18]
13	**Near-field two-photon graphene oxidation**	Femtosecond laser (515 nm)	**Scattering-SNOM**	**Graphene monolayer**	dimensions ranging from ~10 - 30 nm lateral and ~1 nm vertical resolution	**This work**

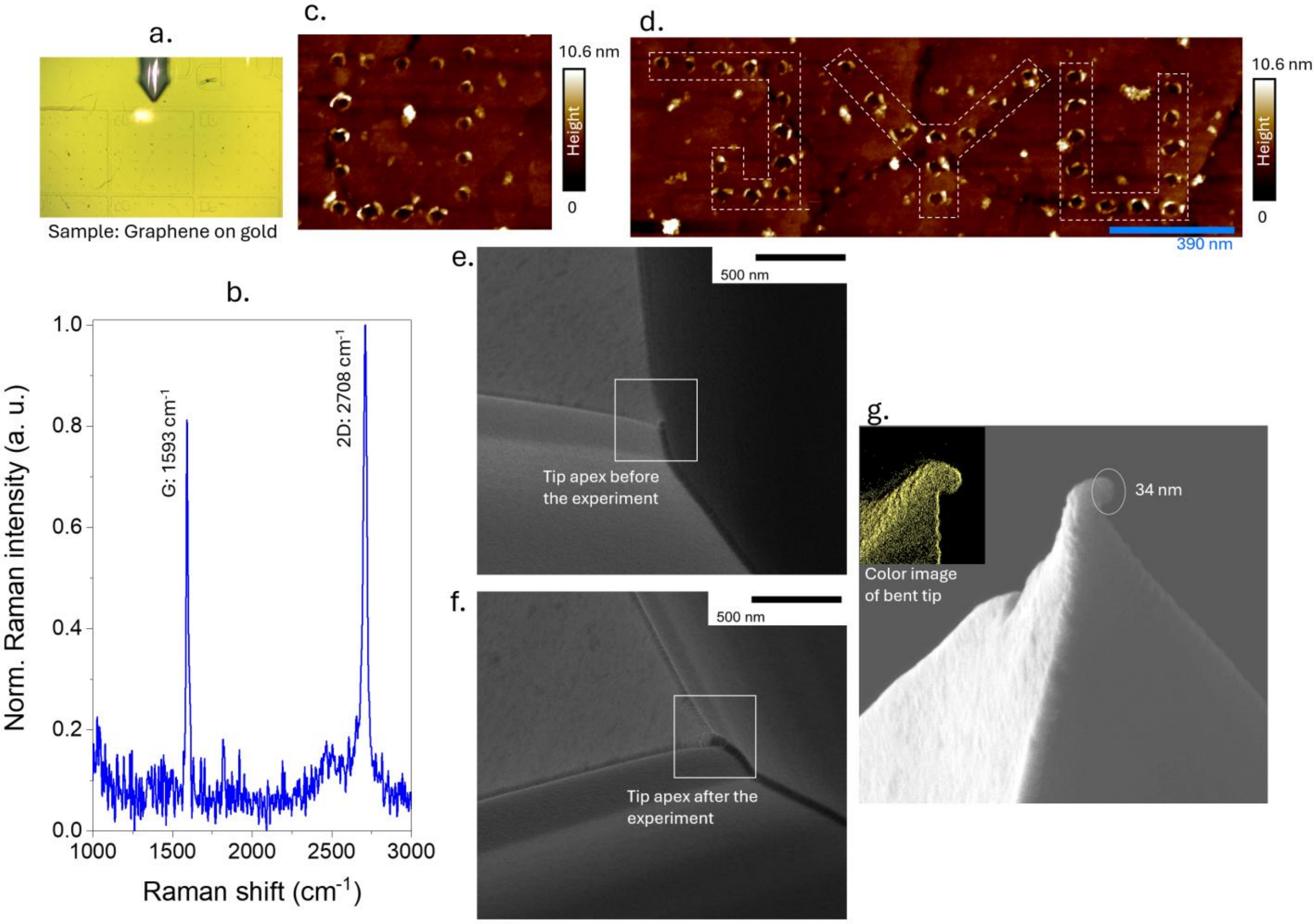

Figure S1: (a) s-SNOM microscopy image of the sample (graphene on gold substrate size: 5 x 5 mm). (b) Raman scattering spectra of the gold/graphene sample. (c)-(d) AFM topography images showing the nanopatterning of an array of nano-punch holes on the graphene surface, forming square and JYU (University of Jyväskylä) shapes, respectively. The scale bar represents 390 nm. (e)-(f) Scanning helium ion microscope images of the SNOM tip before and after the patterning experiment. (g) Magnified image of the tip after the experiment, with the apex measuring around 34 nm. The inset shows a color 3D image of the tip, slightly bent, demonstrating the effects of prolonged contact-mode nanopatterning.

E. Statistical quantification of nano-punch holes:

Table: Details of the measured dimensions of each nano-punch hole

Punch hole	Punch hole depth (nm)	Punch hole size (nm) along the y-axis	Punch hole size (nm) along the x-axis
1st	6.5	41.02	47.35
2nd	7	41.12	47.67
3rd	6.5	41.92	49.81
4th	7.2	43.14	46.91
5th	7	43.03	47.59
6th	7.1	43.69	49.52
7th	6.5	41.44	45.27
8th	7.6	43.74	48.53

Table: Statistical analysis of the dimensions of nano-punch holes

Parameter	Mean (nm)	Std Dev (nm)	Min (nm)	Max (nm)	Range (nm)	CV (%)
Punch Hole Depth	6.93	0.40	6.50	7.60	1.10	5.76%
Size along the y-axis	42.39	1.14	41.02	43.74	2.72	2.69%
Size along the x-axis	47.83	1.46	45.27	49.81	4.54	3.06%

The statistical analysis of the punch hole dimensions provides strong evidence of the precision and reproducibility of our nanopatterning technique. Below is a detailed interpretation of each parameter:

The depth of the punch holes shows moderate variability, with a small range of 1.10nm. The CV of 5.76% indicates that the process is relatively consistent, but there is slightly more variability in depth compared to the horizontal (along x-axis) and vertical (along y-axis) sizes. This could be attributed to subtle variations in laser energy deposition or tip-sample interaction forces during patterning. The vertical size exhibits very low variability, as evidenced by the small standard deviation (1.14nm) and CV (2.69%). This demonstrates excellent control over the vertical dimension of the punch holes, which is critical for applications requiring precise feature sizes. Similar to the vertical size, the horizontal size shows low variability, with a CV of 3.06%. The slightly larger range (4.54nm) compared to the vertical size may reflect minor asymmetries in the near-field enhancement or slight mechanical drift during patterning. However, the overall consistency is still excellent. We can conclude the findings from the statistical analysis in the following way; Precision: The low CV values (<6%) across all parameters indicate high precision and reproducibility in the nanopatterning process.

Consistency: Both the vertical and horizontal sizes exhibit tighter control compared to the depth, suggesting that the technique is particularly well-suited for creating features with consistent lateral dimensions.

Variability Sources: The slightly higher variability in depth (5.76%) may arise from factors such as laser power fluctuations, tip condition, or tip-sample distance variations. Future works can further optimize these factors.

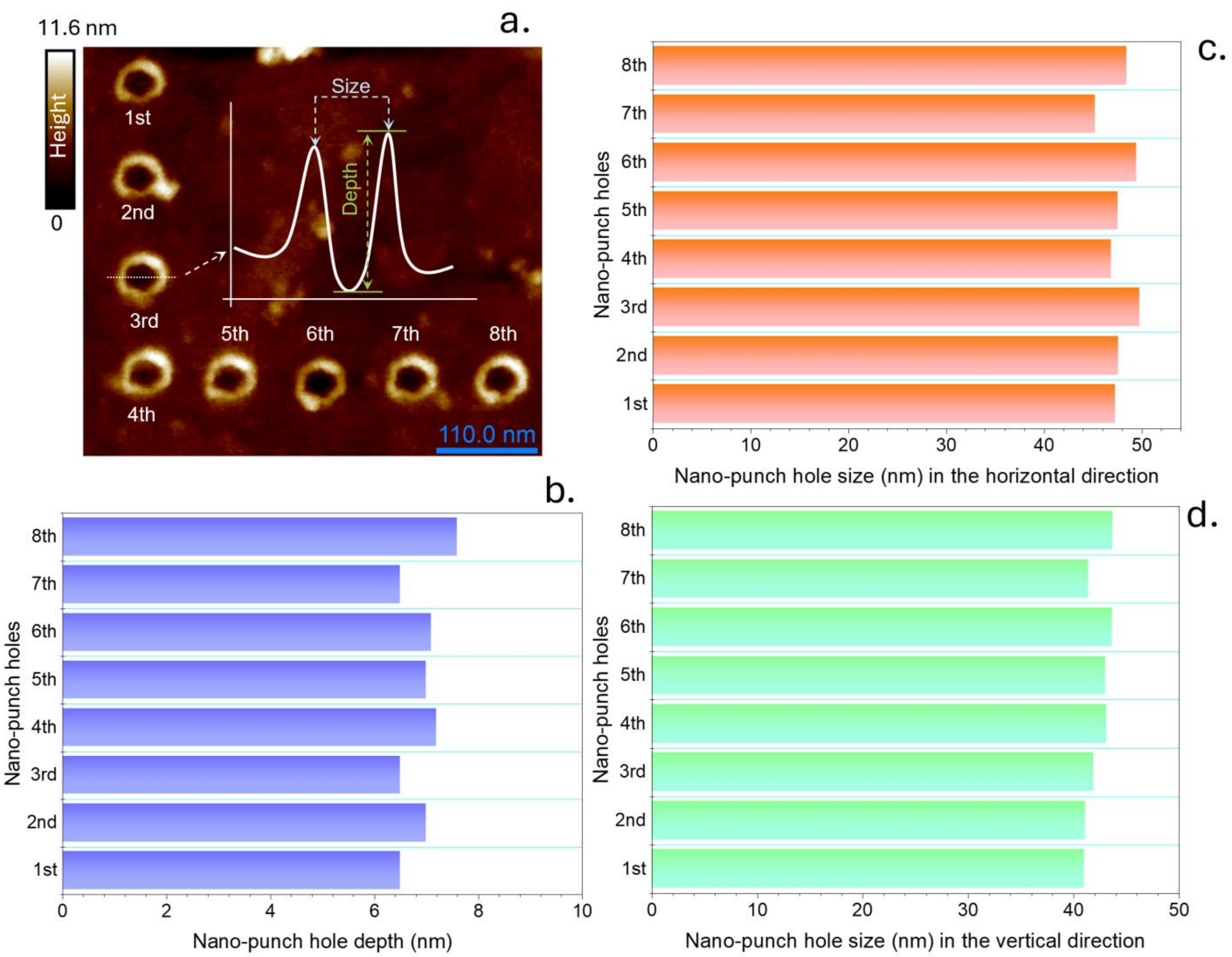

Figure: (a) Atomic force microscopy (AFM) image showing eight individual nano-punch holes on graphene on gold substrate, labeled from 1st to 8th. The color bar indicates height variation. The inset shows the measuring process of punch hole depth and size. (b) Bar chart displaying the punch hole depths in nm scale for each nano-punch hole. (c) The size distribution along the x-axis (horizontal direction) of nano-punch holes shows slight variation across all holes. (d) The size distribution along the y-axis (vertical direction) of nano-punch holes shows an almost consistent hole size with minor fluctuations.

F. Impact of tip degradation on patterning precision:

The experiments were carried out using a tapping-mode AFM tip specifically designed for nano-imaging applications. For future studies, we intend to use a contact-mode AFM tip, as nanopatterning in SNOM is typically performed under contact-mode conditions. We anticipate that employing a tip suited for contact-mode operation will help minimize tip degradation during extended nanopatterning.

Since the tip employed in the present study was not optimized for contact-mode operation, we observed some bending of the tip apex under prolonged use, as confirmed by helium ion microscopy (HIM) imaging. Despite this bending, the dimensions of the tip apex remained largely consistent, as shown in Figure S1, and no signs of tip blunting were observed even after more than 15 hours of continuous nanopatterning.

This consistency in tip condition is also supported by the statistical data presented in Figure 2, which shows minimal variation in nano-punch hole dimensions. However, we did observe one instance (Figure S1c) where the dimensions of the nano-punch holes appeared to be affected by tip bending (based on our assumption). Details of this specific case are provided below.

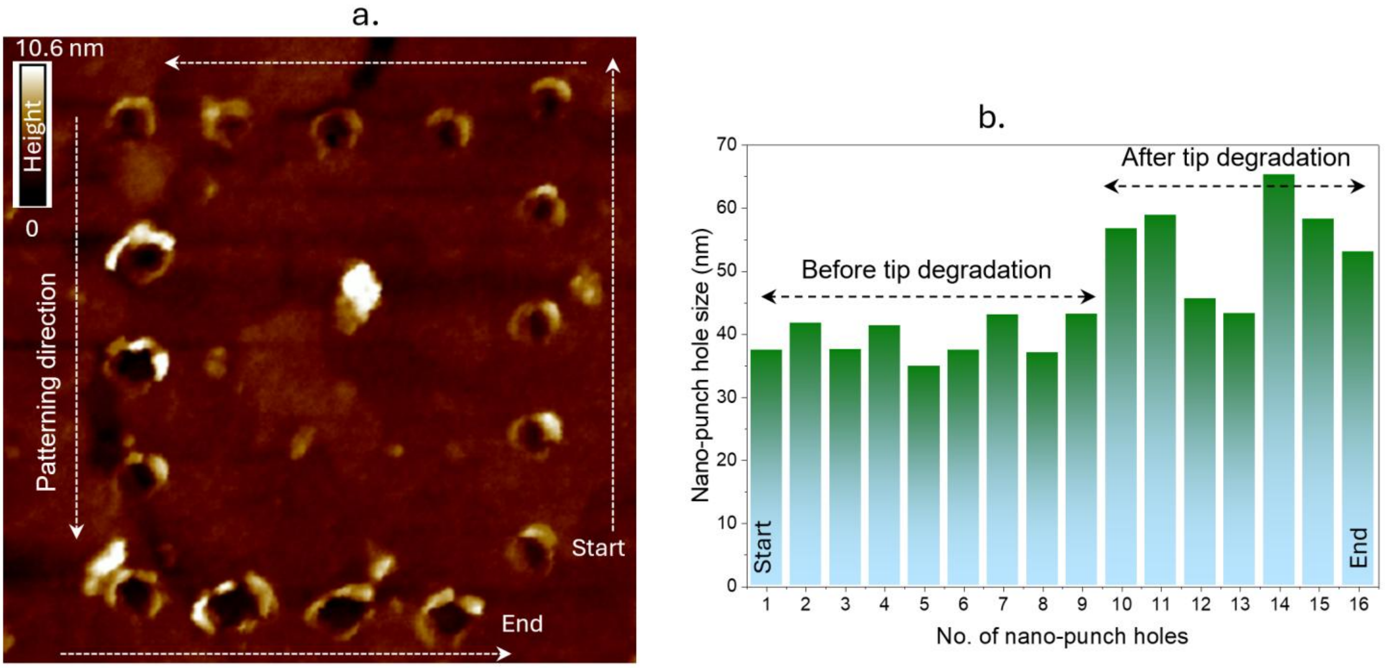

Figure S3: (a). AFM image of nanopatterned holes on a graphene surface. The dashed arrows indicate the direction of nanopatterning, starting from the bottom right and ending up in the same place after the full round. The color scale represents height variation, with brighter areas indicating higher elevations. (b) Bar graph depicting the size of each nano-punch hole created during the patterning process. The graph shows the impact of tip degradation on hole dimensions, distinguishing between the relatively uniform hole sizes before degradation (holes 1–9) and the increased, less consistent sizes observed after tip degradation (holes 9–16).

Table: Details of the measured dimensions of each nano-punch hole

Nano-punch hole	Size (nm)	Nano-punch hole	Size (nm)	Nano-punch hole	Size (nm)	Nano-punch hole	Size (nm)
1	37.43	5	34.91	9	43.10	13	43.28
2	41.75	6	37.46	10	56.72	14	65.31
3	37.49	7	43.00	11	58.87	15	58.24
4	41.28	8	37.00	12	45.55	16	53.06

Statistical analysis of the above data, Mean: 45.90 nm, Standard Deviation: 9.46 nm, Range: 30.40 nm, Coefficient of Variation (CV): 20.60%. The AFM image (a) shows a square-like pattern of nano-punch holes fabricated on a graphene sheet. The patterning begins at the top right and progresses toward the bottom left, as indicated by the dashed white arrows. Initially, the holes appear uniform and well-defined (from 1st to 9th). However, as patterning continues, some holes become distorted or larger, suggesting changes in the tip condition used for patterning. This visual trend is quantitatively supported by the bar graph (b), which plots the size of each nano-punch hole. Holes 1st through 9th show relatively consistent sizes, reflecting stable tip performance. From hole 10th onward, the sizes start to vary more and generally increase, indicating that the patterning tip has undergone degradation.

G. Estimation of laser Intensity and near-field enhancement for two-photon oxidation:

We calculated the far-field peak intensity (I_{peak}) using the following experimental parameters: Pulse duration (τ): 250 fs, beam waist (ω_0): 1 µm, energy per pulse (E_{pulse}): 1.167 × 10^{-9} J, average power (P_{ava}): 0.7 mW, repetition rate (*f*): 600 kHz. This yields an estimated far-field peak intensity of I_{peak} ≈ 2.97 × 10^{11} W/cm², which falls within the accepted range for two-photon oxidation of graphene (~10^{11}–10^{12} W/cm²), as reported by Aumanen et al. [19]. At intensities above ~10^{12} W/cm², ablation becomes dominant.

To estimate the near-field intensity at the tip apex, we consider the following: Laser wavelength (λ): 515 nm, tip material: Platinum-coated, radius (*r*) ~20–25 nm (as per the AFM tip box information), near-field enhancement factor (F_{NF}): ~3.6 (from the electromagnetic simulation of a platinum nanosphere of radius 25 nm at 515 nm). Using these parameters, we obtained conceptual calculation. The incident electric field is approximately, $E_{inc} = \sqrt{\frac{2I_{peak}}{c\epsilon_0}} \approx 1.5\times10^9$ V/m, (here. c = 3×10^8 m/s, ϵ_0 = 8.854×10^{-12} F/m), Local electric field at tip apex, $E_{NF} = F_{NF} \times E_{inc}$ ≈ 5.4×10^9 V/m, Near-field intensity at tip apex, $I_{NF} = \frac{1}{2}c\epsilon_0|E_{NF}|^2$ ≈ 3.8 x 10^{12} W/cm^2. This is the local peak intensity experienced by the graphene surface right under the tip apex. Also, the value of near-field energy density $U_{NF} = \frac{1}{2}\epsilon_0|E_{NF}|^2$ ≈ 1.29×10^8 J/m^3 and fluence (energy per unit area during 250 fs pulse): $I_{NF} \times \tau$ ≈ 0.97 J/cm^2

Therefore, I_{NF} is also in the range for two-photon oxidation of graphene (~10^{11}–10^{12} W/cm²), as reported by Aumanen et al. [19]. This is due to the ultrashort pulse duration (250 fs), which enables non-thermal material processing. Energy is deposited before thermal diffusion occurs, leading to bond breaking and material modification without widespread damage.

H. Clarification of thermal vs. non-thermal effects on nanopatterning:

Our experiments employed a 600 kHz femtosecond pulsed laser (pulse duration: 250 fs; pulse energy: 1.167 nJ). This corresponds to a pulse separation of 1.67 µs. For a 25 nm focal radius (SNOM tip radius), the lateral cooling time follows from the Green-function solution of the 2-D heat–diffusion equation. The time required for heat to spread across the spot itself is, $t_{diff} = \frac{L^2}{4\alpha}$ (thermal diffusion time), L is the thermal diffusion length, and α is the thermal diffusivity. Taking the lowest reported in-plane thermal diffusivity of graphene, α = 1.3 × 10^{-4} m^2 s^{-1} [20], we obtain t_{diff} ≈ 1.2 ps. Using a more typical α = 0.9×10^{-3} m^2 s^{-1} shortens this to t_{diff} ≈ 170 fs. For far field, when L = 1 µm, then the t_{diff} ≈ 65 ps.

pulse separation of 1.67 µs > 1.2 ps to 170 fs (analytical estimation for near-field)
> 65 ps (analytical estimation for far-field)
> 100 ps (literature value; [21])

Therefore, every lattice site is fully relaxed at least 10^5 times before it is re-excited. Under these conditions, the photothermal contribution is negligible, and two-photon photochemical oxidation remains the dominant mechanism. It is to be noted that, during the interpulse gap (Δt = 1.67 µs) the residual heat front expands to, $\sqrt{4\alpha\Delta t}$ ≈ 60 µm. So, the residual heat can spread up to ~60 µm laterally (in-plane) in graphene during the interpulse time of 1.67 µs. This is far larger than the typical laser spot (~1 µm) or s-SNOM feature size (~50 nm). Therefore, any local heating from one pulse is fully diffused into the surrounding graphene before the next pulse hits.

I. Details of the approach curve:

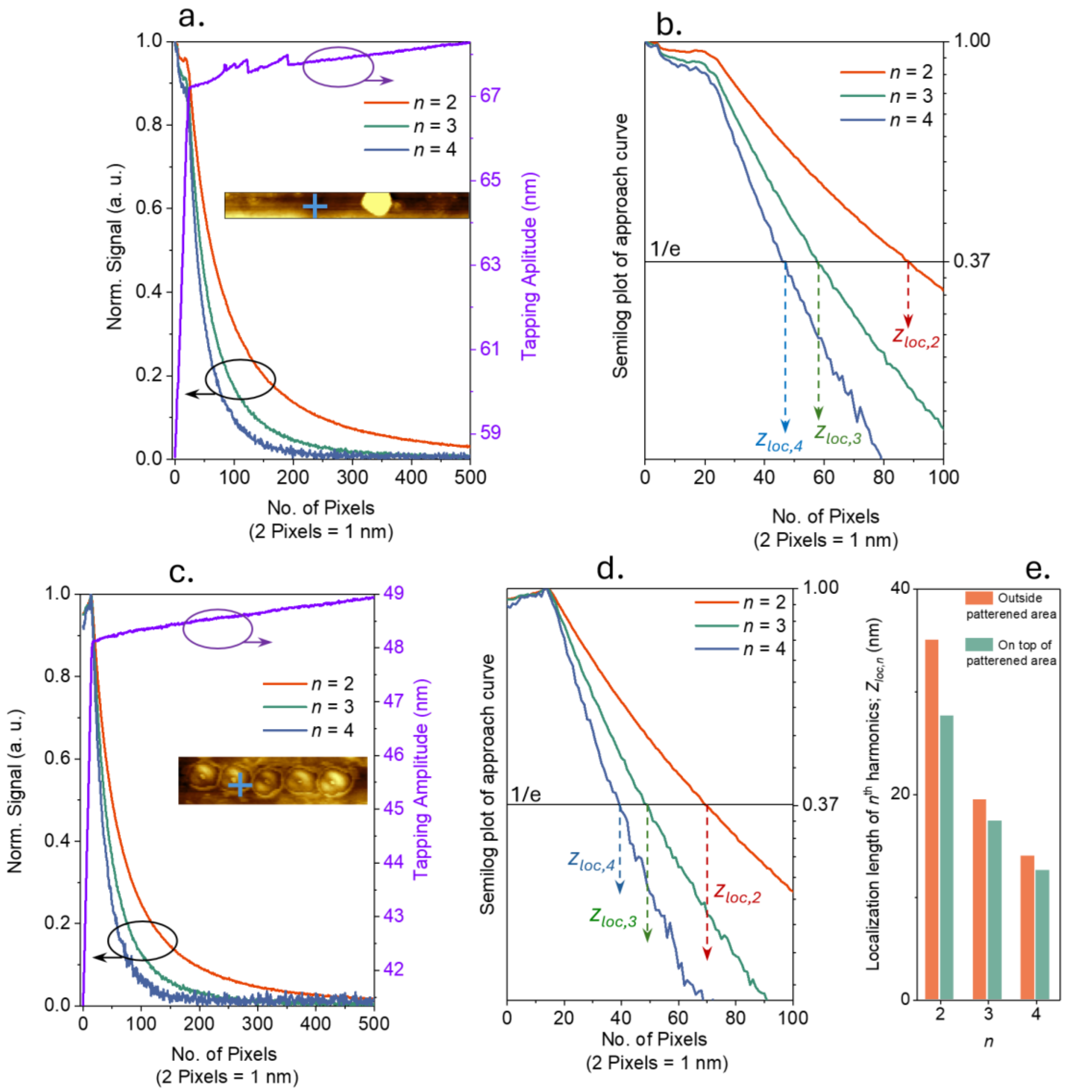

Figure S4: Panels (a) and (c) show the approach curve measured on pristine graphene and nanopatterned region for different harmonic orders (n=2,3,4). Panels (b) and (d) show the localization length ($Z_{loc,n}$; where n = 2, 3, 4 correspond to the 2nd, 3rd, and 4th harmonics) derived from the approach curve in (a), and (c), respectively, for each harmonic. (e) Comparison of the $Z_{loc,n}$ between pristine graphene and the nanopatterned region for the different harmonics.

The approach curves in panels (a) and (c) of Fig. S2 depict the normalized near-field signal as a function of the tip-sample distance, for pristine graphene and nanopatterned regions, respectively. The decay of the signal with increasing distance indicates the dominance of the near-field signal close to the surface. In both pristine and nanopatterned regions, the decay becomes sharper with increasing harmonic order (n=2,3,4), reflecting the reduced localization length of higher harmonics. We have derived the localization length ($Z_{loc,n}$) from approach curves. The localization length quantifies how rapidly the near-field signal decays with distance. It is determined from the semi-log plot of the approach curves, where the distance at which the signal drops to *1/e* of its maximum value is identified. For pristine graphene (panel b), the localization lengths are generally longer compared to the nanopatterned regions (panel d). This suggests that the nanopatterned area enhances near-field coupling due to increased scattering at defects and functionalized edges. Panel (e) compares $Z_{loc,n}$ for the pristine and nanopatterned areas. Across all harmonics, the localization lengths in the nanopatterned regions are shorter, indicating stronger confinement of the near-field signal. Higher harmonics (n=4) exhibit the smallest localization lengths, consistent with their greater sensitivity to nanoscale features and material properties.

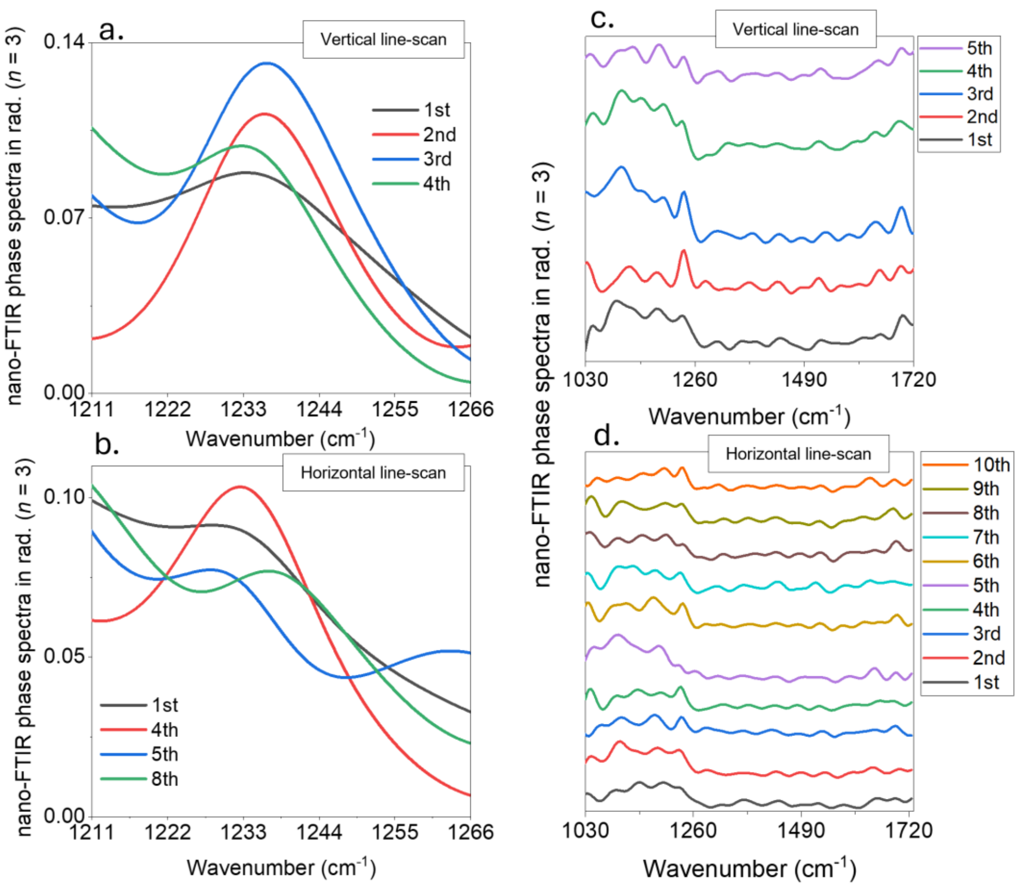

Figure S5: The 1236 cm⁻¹ absorption band region from Fig. 3b and Fig. 3c is plotted separately in (a) and (b) to clearly illustrate the difference in phase values between pristine graphene and the nanopatterned graphene region. (c) and (d) 3rd harmonic Nano-FTIR phase spectrum in the vertical and horizontal direction, respectively, provides the phase data referenced in Fig. 3 of the main manuscript. The vertical and horizontal line scans consist of 5 and 10 spectra; however, only 4 spectra are shown in Fig. 3b and c to maintain their spectral correlation. Here, we present the complete set of spectra recorded during the line scan. For the exact location of the line scan and its corresponding height profile, please refer to Fig. 3.

K. Nanopatterning of graphene on a SiO_2/Si substrate

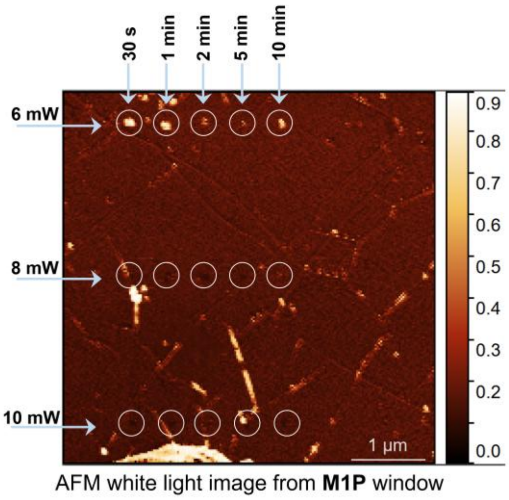

Figure S6: s-SNOM-enabled near-field femtosecond laser nanopatterning of graphene on a SiO_2/Si substrate. The image shown is an AFM mechanical phase map. Laser powers of 6 mW, 8 mW, and 10 mW were used, with exposure times ranging from 30 seconds to 10 minutes (30 s, 1 min, 2 min, 5 min, and 10 min). Laser power values were measured before the laser beam entered the s-SNOM instrument. At higher powers, ablation of the graphene surface was observed, whereas lower powers led to the formation of nanoscale surface structures. It is important to note that the graphene in this sample may have residual polymer on the surface, which could influence the patterning results. In contrast, the graphene-on-gold samples were prepared to ensure no polymer residue on the surface. Therefore, we changed to gold samples, where polymer residues could be avoided. Now, after successful results with gold, we will certainly get back to SiO_2/Si and re-attempt the nanopatterning on it.

References:

[1] F. Huth, A. Govyadinov, S. Amarie, W. Nuansing, F. Keilmann, R. Hillenbrand, *Nano Lett.* **2012**, *12*, 3973.
[2] L. Mester, A. A. Govyadinov, R. Hillenbrand, *Nanophotonics* **2022**, *11*, 377.
[3] X. Chen, D. Hu, R. Mescall, G. You, D. N. Basov, Q. Dai, M. Liu, *Adv. Mater.* **2022**, *34*, e2205636.
[4] F Huth, *PhD Thesis,* CIC NanoGUNE **2015**. (https://www.nanogune.eu/sites/default/files/PhD_thesis_Florian_Huth.pdf)
[5] J. Boneberg, H.-J. Münzer, M. Tresp, M. Ochmann, P. Leiderer, *Appl. Phys. A Mater. Sci. Process.* **1998**, *67*, 381.
[6] J. Boneberg, M. Tresp, M. Ochmann, H.-J. Münzer, P. Leiderer, *Appl. Phys. A Mater. Sci. Process.* **1998**, *66*, 615.
[7] J. Jersch, F. Demming, J. Hildenhagen, K. Dickmann, *Opt. Laser Technol.* **1998**, *29*, 433.
[8] J. Jersch, K. Dickmann, *Appl. Phys. Lett.* **1996**, *68*, 868.
[9] A. Kirsanov, A. Kiselev, A. Stepanov, N. Polushkin, *J. Appl. Phys.* **2003**, *94*, 6822.
[10] Y.-F. Lu, B. Hu, Z.-H. Mai, W.-J. Wang, W.-K. Chim, T.-C. Chong, *Jpn. J. Appl. Phys. (2008)* **2001**, *40*, 4395.
[11] C. Jabbour, J.-L. Lacour, M. Tabarant, A. Semerok, F. Chartier, *J. Anal. At. Spectrom.* **2016**, *31*, 1534.
[12] S. M. Huang, M. H. Hong, Y. F. Lu, B. S. Lukỳanchuk, W. D. Song, T. C. Chong, *J. Appl. Phys.* **2002**, *91*, 3268.
[13] X. Yin, N. Fang, X. Zhang, I. B. Martini, B. J. Schwartz, *Appl. Phys. Lett.* **2002**, *81*, 3663.
[14] A. A. Milner, K. Zhang, Y. Prior, *Nano Lett.* **2008**, *8*, 2017.
[15] A. Chimmalgi, T. Y. Choi, C. P. Grigoropoulos, K. Komvopoulos, *Appl. Phys. Lett.* **2003**, *82*, 1146.
[16] H. Yin, J. Cui, X. Ren, N. Ullah, B. Theogene, Z. Fan, W. Wang, X. Mei, *Surf. Interfaces* **2024**, *46*, 103976.
[17] Y. Lin, M. H. Hong, W. J. Wang, Y. Z. Law, T. C. Chong, *Appl. Phys. A Mater. Sci. Process.* **2005**, *80*, 461.
[18] N. Murphy-DuBay, L. Wang, E. C. Kinzel, S. M. V. Uppuluri, X. Xu, *Opt. Express* **2008**, *16*, 2584.
[19] J. Aumanen, A. Johansson, J. Koivistoinen, P. Myllyperkiö, M. Pettersson, *Nanoscale* **2015**, *7*, 2851.
[20] A. A. Balandin, *Nat. Mater.* **2011**, *10*, 569.
[21] E. Pop, V. Varshney, A. K. Roy, *MRS Bull.* **2012**, *37*, 1273.
